# Association of the use of bacterial cell wall synthesis Inhibitor drugs in early childhood with the Developmental Defects of Enamel

**Published:** 2014

**Authors:** Amna Tariq, Munawar Alam Ansari, Muhammad Owais Ismail, Zahida Memon

**Affiliations:** 1 Dr. Amna Tariq, BDS, Department of Pharmacology, Ziauddin University, Clifton, Karachi, Pakistan.; 2 Prof. Dr. Munawar Alam Ansari, PhD, Department of Pharmacology and Therapeutics, Liaquat University of Medical and Health Sciences, Hyderabad, Pakistan.; 3 Dr. Muhammad Owais Ismail, MPhil, Department of Pharmacology, Ziauddin University, Clifton, Karachi, Pakistan.; 4 Prof. Dr. Zahida Memon, PhD, Department of Pharmacology, Ziauddin University, Clifton, Karachi, Pakistan.

**Keywords:** Molar Incisor Hypomineralization (MIH), Developmental Defects of Enamel (DDE), Hypominerelization

## Abstract

***Objective:*** Our objective of the study was to determine the association between frequent use of Penicillins and Cephalosporins with developmental defects of enamel in pediatric age group.

***Methods:*** This is a cross sectional study, conducted at Ziauddin University. A total of 367 children, having the history of either Penicillin or Cephalosporin exposure were included. The parents of children were asked to complete a questionnaire related to disease and drug history. Dental examination was carried out to assess the hypomineralization in tooth enamel based on modified Developmental Defects of Enamel (DDE) index.

***Results:*** Out of 367 children, 124 (34%) were males and females were 243(66%). In the study group 22.6% (n= 83) of children were found to be hypomineralized. The maximum type of teeth defects were diffused opacities that was 12.0% (n=44). The statistically significant association (p-value < 0.05) was found between frequency of antibiotic use and hypomineralization for most teeth. Children who were exposed to either Penicillins or Cephalosporin in early childhood showed significant (p-value < 0.002) hypomineralized enamel.

***Conclusion:*** This study concludes that frequent use of antibiotics such as penicillins and cephalosporins has positive association with enamel hypomineralization in developing tooth structure.

## INTRODUCTION

Tooth enamel is the most mineralized and hardest tissue in the human body. Its formation is a complex, highly intensive and well-planned biomineralization process.^1^ Enamel develops into the hardest body tissue from 20% by weight to 1% organic material.^2^

The process of amelogenesis is a complex and regulated by ameloblasts that requires secretion of certain matrix proteins that includes amelogenins, amelins, enamelins and tuftelins and the previously formed dentine.^3^ Literature survey revealed that amelogenesis has three phases that are pre-secretory, secretory, and maturation.^3^ A number of genetic and environmental factors influence these processes; consequently, developmental enamel defects (DED) may result from any event disturbing these phases.^4^ Hence the dental crown provides a permanent record of metabolic or systemic alterations during its formation. Systemic disturbances may include recurrent high grade fever, nutritional deficiencies, congenital factors, infections, and certain medications can affect enamel-forming cells.^4^ There are also a number of conditions like infections, metabolic anomalies, and environmental factors that can alter appropriate enamel development.^4^^-^^7^ So, the developmental enamel defects can be defined as any dental enamel alteration resulting from varied disturbances during the process of amelogenesis.^5^

There are only few drugs recognized to alter the normal dental hard tissue formation. Among them are anticancer drugs, such as cyclophosphamide, and antibiotics like tetracyclines, macrolides and sulfonamides influence the teeth development.^8^ Therefore, a small effect on dental enamel could have a significant effect on the public’s dental health because of its extensive use.^1^ According to Beentjes et al it the use of antibiotics is associated with so-called ‘molar incisor hypomineralization’ (MIH)^9^ or with fluorosis-like lesions affecting teeth that mineralize during the first years of life.^10^

A number of studies have shown that the developing enamel is chiefly sensitive to disruptions from external factors and use of antibiotics in early childhood resulting in different types of enamel defects.^4^ Amoxicillin is one of the most common antibiotics that is very frequently used among pediatric patients and has been considered of a low risk drug for infants^11^ but it has been reported in few studies that amoxicillin use is associated with developmental enamel defects^12^ predominantly diffuse opacities. These diffuse opacities, possibly due to enamel hypomineralization, appear clinically somewhat similar to dental fluorosis.^13^

Penicillin and cephalosporin drugs are commonly used in pediatric age group although these drugs are considered as low risk drugs for amelogenesis but few studies have raised questions regarding their safety in above mentioned age group. Our objective of the study was to determine the association between frequent use of penicillin and cephalosporin with developmental defects of enamel in pediatric age group.

## METHODS

This is a cross sectional study, conducted at Ziauddin University after approval by ethical review committee. Informed parental consent was taken prior to study. A total of 367 children were included having an age of 7-14 years from four different schools, located in Federal B area, Karachi. Federal B area is a residential area of Gulberg Town in Karachi, Sindh, Pakistan. There are several ethnic groups residing in Federal B Area including over 99% of the population is Muslim. The population of this town is estimated to be nearly one million. Majority of people living in this area are from the middle socioeconomic status.

The parents of included children were asked to complete a questionnaire related to past disease and drug history. The kids having the history of either Penicillin or Cephalosporin exposure were included in the study.

***Dental examination: ***Dental examination was carried out in four schools of the F.B area. Children were examined in the supine position on the table in medical room of school. Before the examination, each index tooth was cleaned with sterilize gauze in order to remove any gross plaque or food deposits. The teeth were examined ‘wet’. A portable light, disposable mouth mirror and sickle probe was used in the clinical examination. The light source was a fibre-optic examination light, which could illuminate the intra-oral regions during examinations adequately. A probe was used to detect or confirm the presence of any discontinuity of the enamel surface of the teeth. The diagnostic criteria were based on modified Developmental Defects of Enamel (DDE) index defined by the *Federation Dentaire Internationale* (FDI) that is generally used in epidemiological studies. This descriptive index provides information regarding the type (opacity, hypoplasia, and discoloration), number (single and multiple), demarcation (demarcated and diffuses), the demarcated opacities included the white/cream and the yellow/brown subtypes. Under the main type of diffuse opacities, there were subtypes of diffuse lines/patchy, diffuse confluent, confluent/patchy plus staining and/or loss of enamel. Hypoplasia included pits and missing enamel. Twelve permanent erupted teeth were examined including eight upper and lower central and lateral incisors, four upper and lower first molars. A single defect less than 1mm in diameter was not recorded as it is not significant and cannot be justified as hypomineralization on dental examination ground. A tooth was not examined for DDE if less than one third of the tooth surface was visible. Plaque index (PI) was used to rule out the white spot lesions of caries initiation and extent of plaque deposits on tooth surface.

***Statistical analysis: ***The data were entered and analyzed using SPSS (Statistical package for social sciences) version 16. Cross-tabulations were done on selected categorical variables. Associations were subjected to the Chi-square test. Comparisons among frequency to taking medicine groups for the affected teeth children were carried out using analysis of variance. The probability value less than 0.05 was considered significant.

## RESULTS

**Fig.1:**
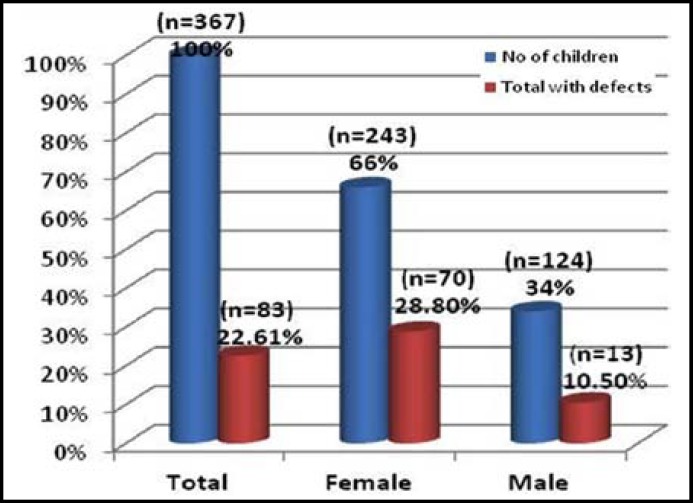
Distribution of children exposed to antibiotic and incidence of teeth defects

A total of 367 students were included in the study, out of which males were 124 (34%) and females were 243(66%) as shown in Fig.1. The mean age was 10.76 ± 1.47 years. In the study group 22.6% (n= 83) of students were found to be hypomineralized.

**Fig.2:**
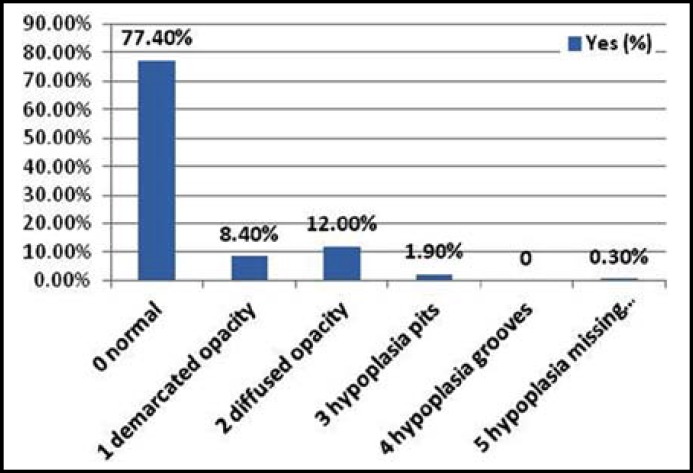
Type of defects on the basis of DDE index

Fig.2 shows the screening results of type of defect according to DDE index and their sub types (diffused and demarcated opacity) in each permanent tooth. The maximum of teeth defects were diffused opacities that was 12.0% (n=44) followed by demarcated opacities, which was 8.4% (n=31) while 77.4% teeth were found to be normal. 

Table-I shows an association between frequency of taking antibiotics and hypomineralization and it reflects the directly proportional relation to hypomineralization for all 12 permanent teeth with increase frequency of taking antibiotics in the past. The statistically significant associations between frequency of antibiotic use and hypomineralization were observed (p-value < 0.05) for most of permanent teeth as shown in this table. However for lower left central incisor, lower left lateral incisor, lower left first molar, lower right central incisor and lower right lateral incisor, p-values could not be calculated as very low percentages of affected teeth were observed against the frequency of taking medicine 

Children who had an exposure of amoxicillin and cephalosporin were found to be hypomineralized, which was 15.4% and 29.2% respectively as shown in Table-II and this association was found to be statistically significant (P-value = 0.002).

## DISCUSSION

The present study investigated the prevalence and possible association of Molar Incisor Hypomineralization (MIH) with the use of antibiotics such as Penicillins and Cephalosporins in a group of children residing in Karachi, Pakistan. To our knowledge there is no published data available on this subject in our part of the world. Literature survey revealed that MIH prevalence varies between studies carried out around the globe. In a study involving children of 5 to 12 years of age in Brazil, approximately 20% of MIH was reported in both dentitions.^14^ Another study conducted in Brazil, including children ages 3 to 5 years found 24.4% MIH.^15^ According to our data MIH prevalence was found to be 22.6% in twelve erupting permanent dentition of children aged between 7-14 years, which is comparable to above mentioned studies. These findings are in accordance to our data may be due to the similarities in the methodological aspects, index and criteria used for the examination of hypomineralization.

A study done in Saudi Arabia by Farsi in 2010 reported 45.4% MIH prevalence in children ages 4 to 5 years.^16^ Another study Conducted by Ramesh et al., in 2010, reported 80% MIH prevalence in 13-year-olds.^17^ Another study done in Iran in 2012 reported 12.7% prevalence of MIH^18^ and much lower prevalence 2.8% has been reported in Chinese children in 2008.^19^ Finding of these three studies are conflicting as compared to our study. These conflicting findings may be due to difference in ethnicity, disease history, socioeconomic status, dietary habits, age of study group and presence of a local pollutant that influence the prevalence of MIH as with the increased age more teeth are erupted that may increase the probability of finding more teeth with MIH^15^ Moreover diagnosis of MIH and its measurements can be manipulated with the type of opacities considered as hypomineralization for the study. Other factors that may mask the hypomineralization assessment is the type of light source used for examination; tooth brushing, prophylactic measurements, drying of teeth before examination or if only anterior teeth or the whole dentition is examined.^15^

**Table-I:** Association between frequency of taking antibiotics and hypomineralization

*No. of teeth affected*		*Frequency of taking Medicines (%)*			*p-value*
		*Less than 3 times*	*Between 3-8 times*	*Greater than 8 times*	
16	Y	2.4	17.7	59.1	< 0.0001
	N	97.6	82.3	40.9	
11	Y	4.4	41.7	59.1	< 0.0001
	N	95.6	58.3	40.9	
12	Y	2.4	33.3	63.6	< 0.0001
	N	97.6	66.7	36.4	
21	Y	4.8	37.5	63.6	< 0.0001
	N	95.2	62.5	36.4	
22	Y	2.4	28.0	60.0	< 0.0001
	N	97.6	72.0	41.0	
26	Y	2.4	17.7	54.5	< 0.0001
	N	97.6	82.3	45.5	
31	Y	0.8	6.2	27.3	N /A
	N	99.2	93.8	72.7	
32	Y	1.2	8.3	27.3	N /A
	N	98.8	91.7	72.7	
36	Y	0.8	7.3	41.0	N /A
	N	99.2	92.7	52.0	
41	Y	0.4	6.2	27.3	N /A
	N	99.6	93.8	72.7	
42	Y	0.8	9.4	18.2	N /A
	N	99.2	90.6	81.8	
46	Y	0.8	7.3	50.0	< 0.0001
	N	99.2	92.7	50.0	

**Table-II:** Association between medicines used and enamel Hypomineralization

	*Outcome*		*p-value*
*Medicine used*	*Normal *	*Hypomineralization*	
Amoxicillin (175)	148 (84.6%)	27(15.4%)	<0.002
Cephalosporin (192)	136 (70.8%	56(29.2%)	

We found significant difference in girls and boys with MIH which may be due to the marked difference in the sample population of males and females in our dataset. These findings are not similar to other studies done in Sweden in the year 2001 and in Rome in 2012 by Jalevik B et al. and Condo R et al. respectively that reported no correlation between sex and incidence of disease.^20^^,^^21^ whereas some authors argue that the frequency of the MIH, in permanent teeth, is greater in the female as compared to males^22^, which is in accordance to WHO Library Cataloguing-in-Publication Data Addressing sex and gender in epidemic-prone infectious diseases 2007, females are more prone to infectious diseases in childhood in South Central Asia as there is low rates of immunization of females as compared to males, that may increase the vulnerability of females towards infections in early age of life and it either directly or indirectly increases the incidence and severity of MIH in females.

It has been observed that childhood infections are very common and for that, kids may expose to common and frequently used antibiotics such as penicillins and cephalosporins during the first few years of life and it has been hypothesized that it put a negative impact on teeth. Our data also supported the same and it was derived that increase frequency of penicillins and cephalosporins use was related to the significantly elevated risk for hypomineralization of early erupting permanent teeth. This may reflect the direct effect of these antibiotics on active ameloblasts or may be attributed to the infectious disease for which antibiotic was prescribed.^8^^,^^12^^,^^23^

The enamel matrix proteins provide the framework for mineralization and play a pivotal role in nucleation, crystal orientation, and crystal growth.^24^ Our results showed that amoxicillin use early during the first year of life seems to be linked to fluorosis-like enamel defects on maxillary central incisors. Considering the developmental stages of enamel formation of maxillary central incisors, it has been hypothesized that amoxicillin may interferes at secretory stages of amelogenesis or may reduce gene expression of matrix proteins (such as amelogenins) or decrease the activity of proteinases that hydrolyze matrix proteins.^2^ However, the exact molecular mechanism(s) of action is still unclear. Furthermore, the cause of developmental enamel defects, such as malnutrition and calcium deficiency, could contribute to the development of this type of enamel anomaly.^25^

## CONCLUSION

It can be concluded that frequent use of bacterial cell wall synthesis inhibitor drugs (Penicillins or Cephalosporins) have strong association with hypomineralization in developing tooth structure. If prescribed in early childhood, it may carry some undocumented risk to the developing teeth. We found MIH prevalence of 22.6% in our study that reflects the high percentage of enamel defect in our sub set of population and it may present as a frequent public health problem that is subjected to optimal prevention, timely diagnosis and proper management. 

Nevertheless, this cross-sectional study provided baseline data in Pakistani population and it is recommended that prospective longitudinal observational studies should be conducted on larger cohort to state the pathophysiological plausibility, biological nature, etiologic factors, and the temporal sequencing of such potential relationship. 

## Notes

***Source of funding:*** Self funded project.
